# The distribution of large floating seagrass (*Zostera marina*) aggregations in northern temperate zones of Bohai Bay in the Bohai Sea, China

**DOI:** 10.1371/journal.pone.0201574

**Published:** 2019-03-12

**Authors:** Xu Min, Zhou Yi, Song Xiao-Jing, Zhang Yun-Ling, Zhang Hai-Peng

**Affiliations:** 1 Key Laboratory of East China Sea and Oceanic Fishery Resources Exploitation, Ministry of Agriculture, Shanghai, China; 2 East China Sea Fisheries Research Institute, Chinese Academy of Fishery Sciences, Shanghai, China; 3 CAS Key Laboratory of Marine Ecology and Environmental Sciences, Institute of Oceanology, Chinese Academy of Sciences, Qingdao, China; 4 Hebei Provincial Research Institute for Engineering Technology of Coastal Ecology Rehabilitation, Tangshan, China; 5 Hebei Ocean and fisheries science research institute, Qinghuangdao, China; Tanzania Fisheries Research Institute, UNITED REPUBLIC OF TANZANIA

## Abstract

Seagrass meadows (*Zostera marina*) are important coastal ecosystems with high levels of productivity and biodiversity. They often have high biomass turnover and are susceptible to dislodgment, leading to export of biomass from seagrass beds. In September 2016, two cruises covering a total area of 52 km x 15 km (38°57'1.14"–39° 0'41.28" N, 118°45'23.22"–118°47'6.96" E) found floating *Z*. *marina* aggregations along 13 km of the transect in the northernmost area of Bohai Bay, in the Bohai Sea, China. This floating seagrass was 6.3–13.4 km northeast (offshore) of the Caofeidian seagrass bed, which is a large (10 km^2^) seagrass bed discovered in 2015 in the Bohai Sea, China. The modal length of floating intact shoots (from meristem to longest leaf tip) matched samples from the Caofeidian seagrass bed. The dominant individuals lengths were 40–50 cm, with less than 5% of the total number of individuals found in larger size categories (80–90 and 90–100 cm). We concluded that they originated from the nearby Caofeidian seagrass meadows.

## Introduction

Seagrass meadows are highly biodiverse and productive coastal ecosystems, covering approximately 0.15% of the global ocean area and contributing 1% of the marine net primary production [1]. Seagrass meadows are important nursery habitats for many economically valuable fish and invertebrate species such as red drum (*Sciaenops ocellatus*), Atlantic cod (*Gadus morhua*), queen conch (*Strombus gigas*), and blue crab (*Callinectes sapidus*) [1–2]. They also provide suitable substrate for a variety of marine epiphytes and facilitate the sedimentation of suspended particulates [3–4]. In the past half century, seagrass meadows have declined rapidly worldwide. For example, approximately 2.6 × 10^4^ km^2^ of seagrass meadows, accounting for 15% of the estimated global total, disappeared between 1993 and 2003 [5].

Floating seagrass aggregations are an important method of transport for seagrass detritus from one habitat to another [6]. As seagrass meadows age and interact physically with the environment around them, leaves may break off from the beds and float to the sea surface [6]. Due to the high primary productivity of seagrass meadows [7], considerable volumes of leaves are continually shed and transported away from the beds by currents and wave action [8–10]. Large aggregations of this floating vegetation called “seagrass wrack” can form [11] and the biomass is ultimately exported to the seafloor or washed ashore on beaches.

Floating at the sea surface, seagrass wracks can serve as habitat “hot spots” similar to other floating vegetation such as floating macroalgae (e.g. *Sargassum horneri* in the East China Sea)[12–14]. Thresher [15] is one of the only studies to report on the trophic impacts of floating seagrass wracks. Through indirect lines of evidence, they reported that microbial decomposition of floating seagrass played a pivotal role in the coastal planktonic food chain [15].

A broad seagrass meadow (39°00’N–39°05’N, 118°41’E–118°44’E), covering approximately 10 km^2^ was discovered in the northwestern area of Longdao Island, Caofeidian, Bohai in October 2015 [16]. To date, it is the largest seagrass meadow discovered in the Bohai Sea and Yellow Sea areas in China, and its composition is dominated by *Zostera marina* [16]. *Z*. *marina* is a species of seagrass known by the common names ‘common eelgrass’, and is widely distributed in the Northern Hemisphere. The plant is a rhizomatous herb which produces a long stem with hair-like green leaves. The rhizome grows horizontally through the substrate, anchoring via clusters of roots at nodes. In September 2016, a large area of floating *Z*. *marina* aggregations was observed in the area of Xiang-yun Island, the northernmost area of Bohai Bay in the Bohai Sea, China, nearby to the Caofeidian area. The objective of the present study was to identify the spatial distribution of floating *Z*. *marina* rafts in northern temperate zones of Bohai Bay in the Bohai Sea, China.

## Materials and methods

The field survey was approved by the Institute of Oceanology, Chinese Academy of Sciences.

**Fig 1:**
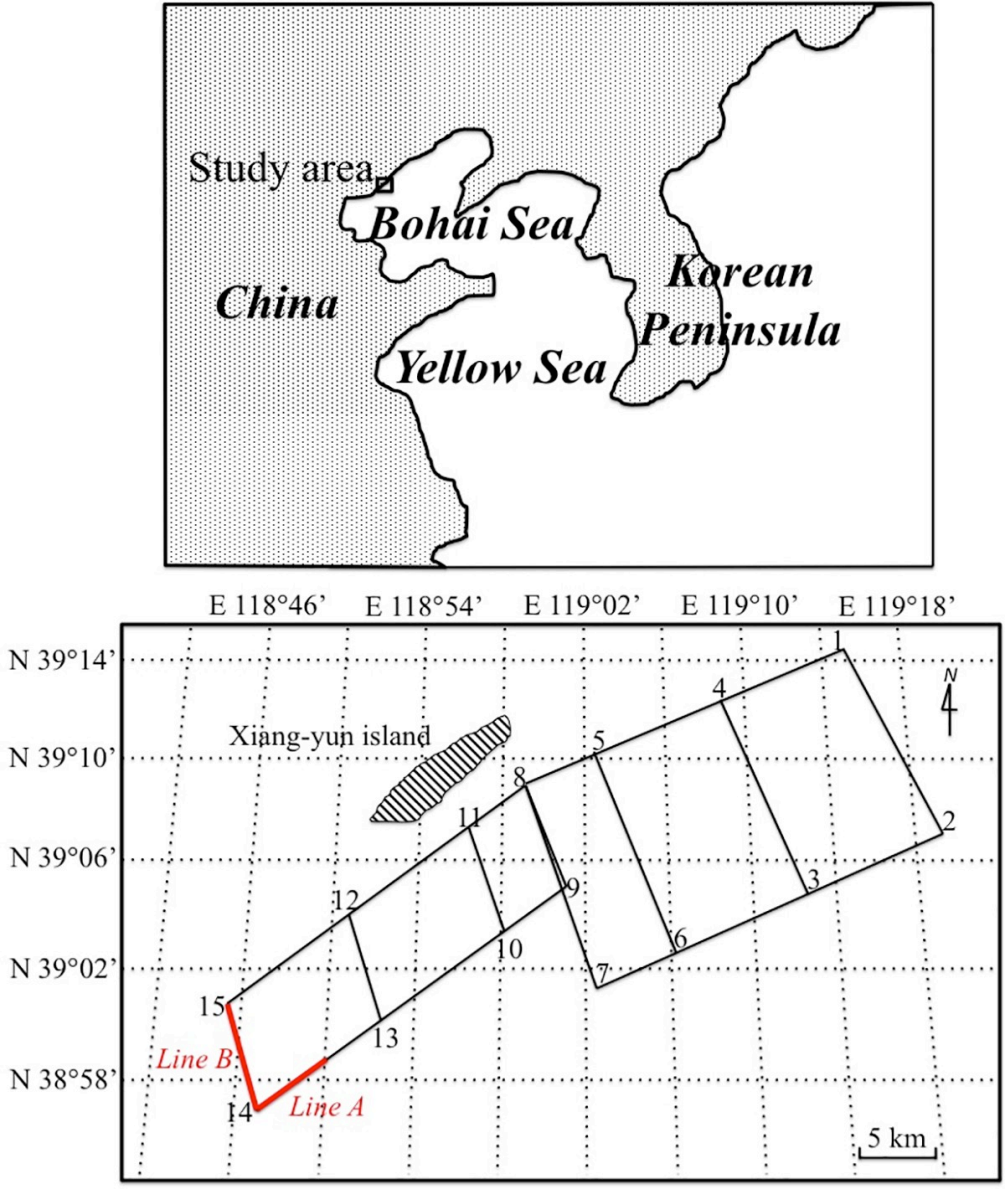
Schematic map showing the route of two research cruises to survey the distribution of floating *Zostera marina* aggregations in the waters off Xiang-yun Island, Tangshan in the northernmost area of Baihai Bay in the Bohai Sea, China. On 6 and 13 September 2016 the vessel travelled from ‘Point 1’ to ‘Point 8’ and from ‘Point 8’ to ‘Point 15’, respectively. The red lines (‘Line A’ and ‘Line B’) indicate the locations of floating *Z*. *marina* aggregations. Line A was from N 38°58.9358’ E 118°50.1150’ to ‘Point 14’, and Line B was from ‘Point 14’ to ‘Point 15’. The Caofeidian seagrass meadows are located approximately 6,400 m away from Point 14, 10,000 m away from Point 15 and 13,500m away from N 38°58.9358’ E 118°50.1150’.

**Fig 2:**
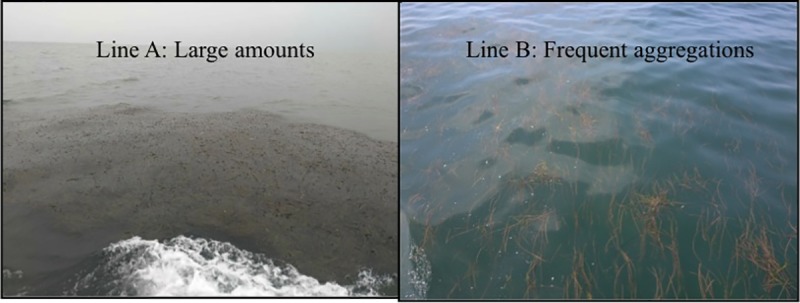
Left: An example of ‘large amounts’ of *Zostera marina* observed in ‘Line A’. Right: An example of floating *Z*. *marina* leaves observed in ‘Line B’.

A survey of the distribution of floating *Z*. *marina* around Xiang-yun Island, the northernmost part of Bohai Bay, Bohai Sea, was carried out using transect lines at a distance of approximately 0.5–1.0 km (Fig 1). Sightings of drifting aggregations are difficult unless the sea is calm, and only aggregations within 50 to 100 m of the vessel were observed. The first cruise was made on 6 September 2016 from ‘Point 1’ to ‘Point 8’, while the second cruise was made on 13 September 2016 from ‘Point 8’ to ‘Point 15’ (Fig 1). The research vessel kept a stable speed of six knots, and seagrass aggregations seen within each transect line were quantified as follows: none, frequent aggregations and large amounts (Fig 2).

Based on previous literature [17–18], a dip net with a ring diameter of 50 cm and mesh size of 5.0 cm was selected for optimal sampling. The net was dipped under each clump, hauled up in one quick sweep whenever possible, and emptied into a bucket containing seawater. Usually collections were carried out when *Z*. *marina* accumulated in large aggregations, while small fragments were ignored. In the laboratory, the samples were rinsed and sorted, keeping together any leaves that remained connected within a single leaf sheath (individual ramet). *Z*. *marina* individuals lengths were measured from the base to the top of the longest leaves (± 1 cm) using a 30 cm ruler. In most cases these samples were broken fragments of leaves, which may have originated from leaf turnover or due to breakdown in the ocean. In a few cases, bundles of leaves with the meristem were attached to a piece of rhizome, indicating that the shoot had been uprooted. These leaves were also often broken at the tips and so shoot length measurements may be an underestimate. The majority of samples for shoot length measurements were composed of leaf fragments, and some were broken leaves with the meristem attached to a piece of rhizome or a couple of broken leaves with only a piece of rhizome (further details are provided in supplementary materials ‘dataset’). We collected 220 and 204 individuals from Point 14 and between N 38°58.9358’ E 118°50.1150’ (Line A) and Point 14 and 15 (Line B) respectively. Then we selected the relatively intact shoots and measured their lengths. For Line A, we measured 53 individuals and 50 individuals for Line B. We used these lengths to build a frequency distribution of plant size, keeping in mind that most leaves were broken and thus underestimate the shoot size from which they came. We also calculated % uprooted as the number of samples with an attached meristem relative to total measured samples (further calculation details are provided in supplementary materials ‘dataset’).

## Results and discussion

**Fig 3:**
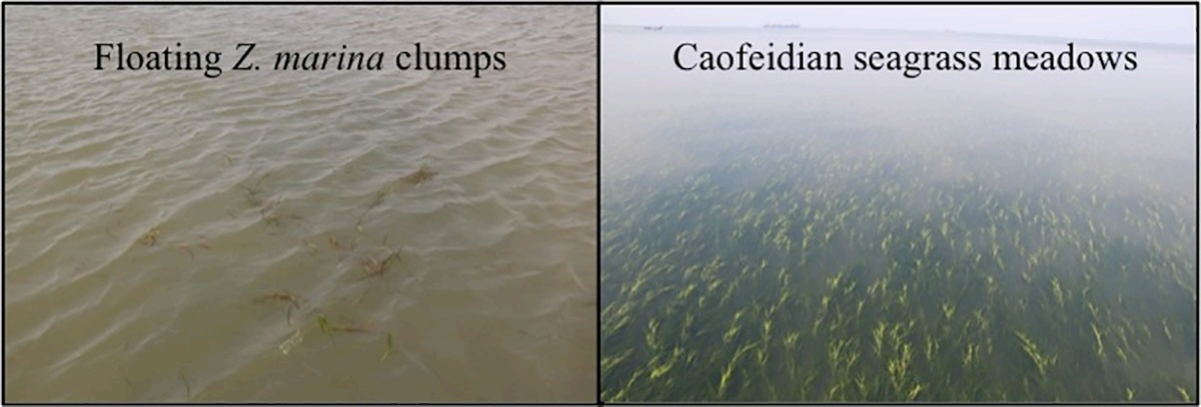
**Left: Floating *Zostera marina*** aggregations **found in Caofeidian seagrass meadows; Right: ~10 km**^**2**^ **Caofeidian seagrass meadows observed on June 1**^**st**^ **2018**.

A large area of floating *Z*. *marina* aggregations was observed in our survey (marked in red, Fig 1.). The area between N 38°58.9358’ E 118°50.1150’ and ‘Point 14’ had frequent aggregations, and the area between ‘Point 14’ and ‘Point 15’ had large amounts of floating aggregations. The fraction of uprooted samples found in the area between N 38°58.9358’ E 118°50.1150’ and ‘Point 14’, and the area between ‘Point 14’ and ‘Point 15’ were 13.21% (7 of 53) and 2.00% (1 of 50), respectively. In the latter transect line, we estimated each aggregations to make up a distance of approximately 80–100 m. The Caofeidian seagrass meadows are located approximately 6,400 m away from Point 14, 10,000 m away from Point 15 and 13,500 m away from N 38°58.9358’ E 118°50.1150’. We also observed ~10 km^2^ seagrass meadows and large scale areas of floating *Z*. *marina* aggregations around an artificial oil platform in the Caofeidian Sea areas, near Long Island (Fig 3) in June 2018. Thus, we suggested that these floating seagrass aggregations originated from the Caofeidian seagrass meadows. Natural disturbances such as weather, tides and the degree of bed exposure, as well as anthropogenic impacts such as dredging, fishing, and anchoring can destroy seagrass meadows and displace plants from the beds to distant locations offshore [8–9, 19–22]. Dierssen et al. [11] found that strong southerly winter winds in Greater Florida Bay advected considerable amounts of seagrass wracks, which comprised predominantly of *S*. *filiforme* from the dense meadows in the area to the oligotrophic Atlantic Ocean Waters. In our study, isolated *Z*. *marina* leaves fragments became more aggregated into patches between ‘Point 14’ and ‘Point 15’. Collections of seagrass aggregations are common in coastal waters due to slow counter-rotating vortices [23].

Generally, floating seagrass rafts can be considered a relatively long-lived ecosystem “hot spot”, providing habitats for a wide variety of marine organisms [24]. The diverse community supported by floating wracks also serves as a resource for larger pelagic fish, which feed on the smaller organisms living on and near the wracks. For example, the floating seagrass debris provides plentiful nutrition and is an important natural food source for an economically important local aquaculture species, the sea cucumber *Apostichopus japonicas*. Song et al. [25] found that the specific growth rates, food utilization efficiencies and energy budgets of *A*. *japonicas* were strongly influenced by the ratio of *Z*. *marina* in their diets. The floating seagrass aggregations observed in this study is close to a sea cucumber marine ranching area; thus the deposited seagrass leaves fragments may serve as a natural diet for this aquaculture species.

**Fig 4:**
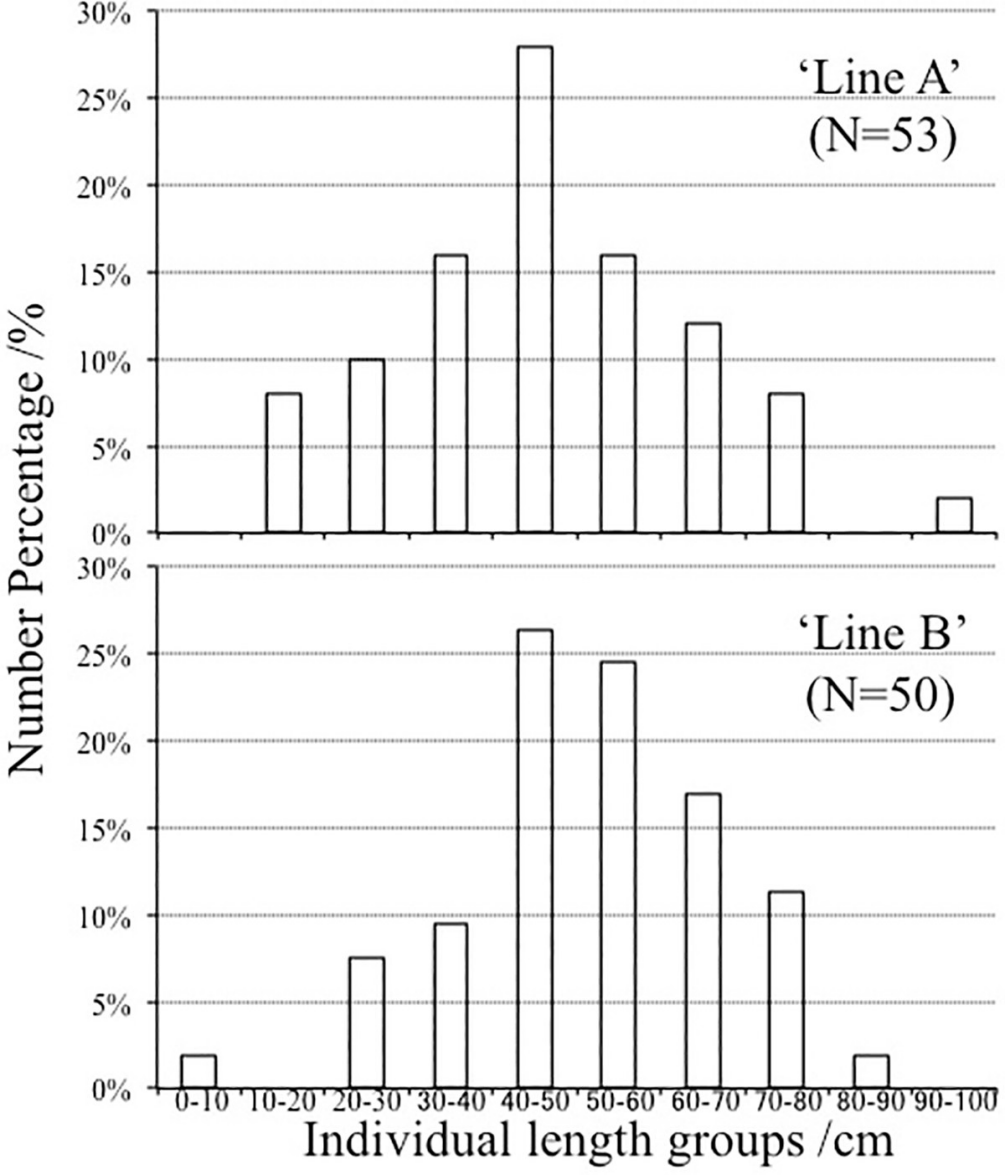
Number percentage (%) of floating *Zostera marina* leaves within different individual length categories (cm) in ‘Line A’ (N = 53) and ‘Line B’ (N = 50).

The dominant individuals length category of the isolated *Z*. *marina* leaves fragments in the present study was 40–50 cm in both ‘Line A’ and ‘Line B’ (Fig 4). In ‘Line A’, > 15% of individuals were in each of the 30–40, 40–50 and 50–60 cm categories. Whereas, in ‘Line B’, > 15% of individuals were in each of the 40–50, 50–60 and 60–70 cm categories. Less than 5% of individuals in both Lines were in the largest size categories of 80–90 or 90–100 cm. In the Caofeidian *Z*. *marina* seagrass meadows, the canopy height was 15.2 ± 5.84 to 62.1 ± 7.34 cm (means ± standard deviation)[1].

This study is the first to report on floating *Z*. *marina* aggregations in the temperate zones along the Chinese coastline of the Bohai Sea. We concluded that the floating seagrass aggregations in the northernmost area of the Bohai Bay (38°57'1.14"–39° 0'41.28" N, 118°45'23.22"–118°47'6.96"E) originated from the Caofeidian seagrass meadows. Our findings provide a greater understanding about the life cycle of *Z*. *marina* including how it is transported to the seafloor and become organic debris for benthic communities. The dislodgement and break-up characteristics of the floating *Z*. *marina* aggregations and the transportation of their organic matter to the seafloor will be the subject of future studies.

## Supporting information

S1 File Dataset. Included all the data used in the paper.
(XLSX)
